# p72 antigenic mapping reveals a potential supersite of vulnerability for African swine fever virus

**DOI:** 10.1038/s41421-024-00692-x

**Published:** 2024-07-31

**Authors:** Qi Yu, Dening Liang, Wangjun Fu, Li Zhang, Jinglin Wang, Zhenjiang Zhang, Yao Sun, Dandan Zhu, BinYang Zheng, Ling Zhu, Ye Xiang, Dongming Zhao, Xiangxi Wang

**Affiliations:** 1 CAS Key Laboratory of Infection and Immunity, National Laboratory of Macromolecules, Institute of Biophysics, Chinese Academy of Sciences, Beijing, China; 2 University of Chinese Academy of Sciences, Beijing, China; 3 Department of Vaccine Clinical Evaluation, Jiangsu Provincial Center for Disease Prevention and Control, Nanjing, Jiangsu China; 4 Yunnan Tropical and Subtropical Animal Viral Disease Laboratory, Yunnan Animal Science and Veterinary Institute, Kunming, Yunnan China; 5 State Key Laboratory for Animal Disease Control and Prevention, National African Swine Fever Para-reference Laboratory, National High Containment Facilities for Animal Diseases Control and Prevention, Harbin Veterinary Research Institute, Chinese Academy of Agricultural Sciences, Harbin, Heilongjiang China; 6 Beijing Advanced Innovation Center for Structural Biology, Beijing Frontier Research Center for Biological Structure, Center for Infectious Disease Research, Department of Basic Medical Sciences, School of Medicine, Tsinghua University, Beijing, China

**Keywords:** Electron microscopy, Molecular biology

Dear Editor,

African swine fever (ASF), first discovered in Kenya in 1921, is an acute hemorrhagic viral disease with a high mortality rate of nearly 100% caused by the African swine fever virus (ASFV)^1^. Over the past few decades, ASFV has spread to many countries and regions, which led to huge economic loss to the swine industry^2^. ASF is designated as a re-emerging viral disease by the World Health Organization and there is no vaccine or treatment available^3^.

ASFV possesses a multilayered structure including a genome-containing nucleoid, the core shell, an inner lipid envelope, an icosahedral protein capsid and an external envelope^4,5^. The outer icosahedral capsid is built from 8280 major capsid proteins (p72) and 60 penton proteins (H240R), organized into 12 pentasymmetrons and 20 trisymmetrons. Three copies of p72 and five H240R molecules constitute a pseudo-hexameric capsomer and a pentameric capsomer, respectively. There are 2760 pseudo-hexameric capsomers and 12 pentameric capsomers arranged in a triangulation number (T) = 277 icosahedral lattice (h = 7 and k = 12)^6^. p72 is the most abundant component of the external icosahedral protein capsid, accounting for over 33% of the total mass of the virus^7–9^.

p72 is one of the major protective antigens since monoclonal antibodies were shown to neutralize virulent ASFV isolates^10^. Although linear antigenic epitopes were reported^11^, there is no structural information available for the p72 epitope so far. To identify the epitopes of p72, analyze the characteristics of antibodies and explore the neutralization mechanism, we used flow cytometry to sort plasmablasts from peripheral blood mononuclear cells of five ASFV-infected swine. Enzyme-linked immunospot assays demonstrated that nearly half of these cells secreted antibodies binding p72. We successfully amplified 59 IgG sequences from single-cell cDNA, then produced recombinant mAbs via transfection into 293T human embryonic kidney cells. Comparison of the length of complementary-determining regions (CDRs) of 59 porcine IgG antibodies and those of human origin revealed that HCDR3 (~15.4 residues) from porcine is ~10% shorter than its counterparts (~16.2 residues) from human; (Supplementary Fig. S1 and Table S1). HCDR2 (~9.7 residues) and LCDR1 (~9.8 residues) from porcine are ~25% and 32% longer than those of human (HCDR2 (~7.8 residues) and LCDR1 (~7.4 residues)), respectively, while HCDR1, LCDR2 and LCDR3 display similar in length (Supplementary Fig. S1). 9 porcine antibodies were selected for structural and functional investigations. Amino-acid sequence alignment of the variable region of 9 Fabs revealed two and three groups for light and heavy chains, respectively (Supplementary Fig. S2). Of note, relatively lower similarity (~40%) is clearly observed between the two groups in light chains. Based on combinations of various groups of light and heavy chains, we divided these 9 antibodies into four classes: I) B1, H5, D7 and E5; II) F11 and F5; III) C9 and H3; IV) G6 (Fig. 1a; Supplementary Fig. S2). Class I antibodies exhibited the highest binding affinities up to 2 nM and classes III and IV showed nearly 1000-fold decreased binding capabilities (Fig. 1a; Supplementary Fig. S3a). F11 and F5 displayed a 400-fold difference in binding activity, presumably owning to key residue substitutions in their paratopes (Y51 in F11; G51 in F5). The competitive surface plasmon resonance (SPR) showed that the association of any of the nine antibodies to p72 inhibited the binding of others, albeit with the presence of partial competition for three antibodies due to their quick dissociation (Fig. 1a; Supplementary Fig. S3b).

**Fig. 1:**
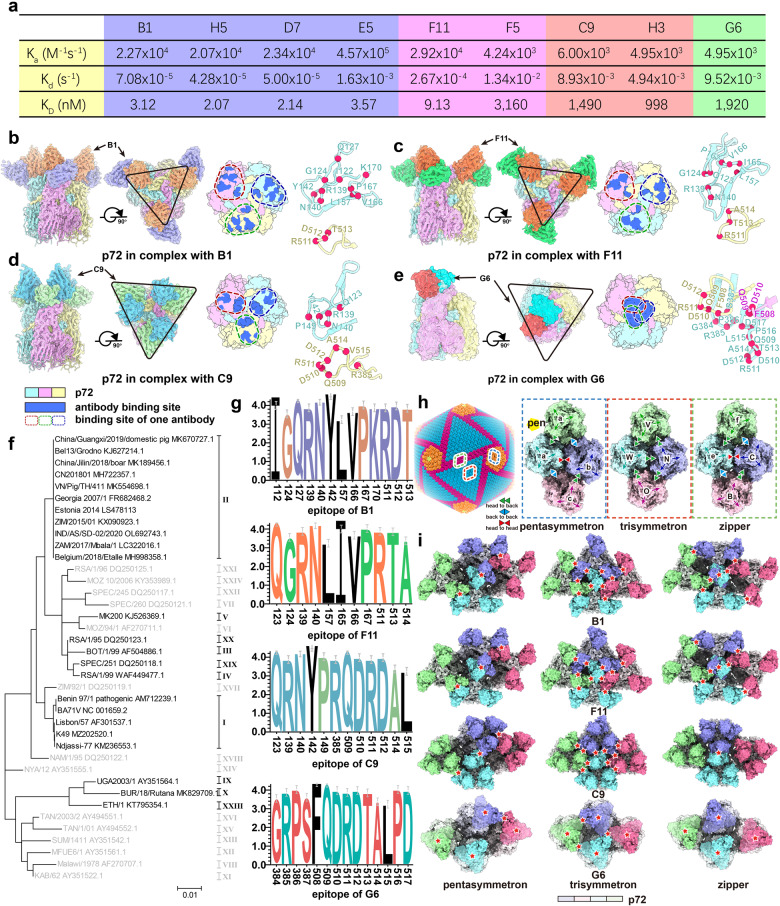
Structural and functional characterization of Fab fragments B1, F11, C9, and G6. **a** Summary of SPR-based analysis of the affinity kinetics of the nine antibodies. **b**–**e** Side and top views (left panel) of cryo-EM maps of p72 in complex with B1 (**b**), F11 (**c**), C9 (**d**) and surface of p72 in complex with G6 (**e**). Surface represents interaction region between p72 and antibodies on p72 (middle panel). Dark blue areas display antibody binding site. The dashed box represents the binding site of one antibody. Right panels show residues involved in the interaction with antibodies. **f** Evolutionary relationships of 38 ASFV isolates based on p72 C-terminal end gene sequences. The strains with light gray lack the full p72 sequence. **g** Sequence conservation analysis of the epitopes of four antibodies on p72. **h** Cryo-EM reconstruction of the ASFV capsid. The trisymmetrons, pentasymmetrons and zippers (the boundaries of two neighboring trisymmetrons) are colored in blue, orange and pink, respectively. The dashed box shows the detailed depictions of three distinct assembly patterns according to their locations: in the pentasymmetron (left), trisymmetron (middle) and zipper (right). Three types of interaction mode: “head to back”, “back to back” and “head to head” are indicated by green, blue and red triangles, respectively. **i** Possible locations of antibody binding in different organizations. The clash between antibodies was marked with red star.

To define the epitopes of antibodies and expound the characteristics and the neutralization mechanism of the antibodies via structural analysis, we determined cryo-EM reconstructions of five antibodies (B1, F11, C9, H3, G6) in complex with p72 at resolutions of 2.75 Å, 2.98 Å, 3.48 Å, 3.24 Å and 4.36 Å, respectively (Fig. 1b–e; Supplementary Figs. S4, S5 and Table S2). To our knowledge, this is the first instance of structures of porcine antibodies, which adopt a classic IgG fold as observed in structures of human antibodies with distinct conformations in CDR regions (Supplementary Fig. S6). All four classes of antibodies recognize conformational epitopes that comprise 2–3 copies of p72 monomers, locating on these exposed regions (ERs) (Fig. 1b–e; Supplementary Fig. S7).

Except for G6, all other antibodies have three copies of Fabs bound to one p72 capsomer, where three B1 and F11 bind on the triangular exterior of the p72 capsomer, constructing three vertexes of the “triangle”; three copies of C9 and H3 associate with the triangular interior surrounding the three-fold axis, occupying three sides of the “triangle” (Fig. 1b–d; Supplementary Fig. S8). Unsurprisingly, C9 and H3 display a similar mode of binding (Fig. 1d; Supplementary Fig. S8). As opposed to these, only one G6 is inserted into the central “groove” of the three-fold axis formed by three p72 monomers (Fig. 1e; Supplementary Fig. S9). Correlated with targeting site, binding of one G6 Fab sterically hinders attachments of other G6 to the p72 capsomer (Fig. 1e). Compared to C9, B1 and F11 exhibit a ~10° clockwise rotation, whilst G6 shows a ~30° counterclockwise rotation upon binding to p72 capsomer (Supplementary Fig. S10). These observations are largely consistent with results of antibody classification and binding assays (Fig. 1b–d).

By analyzing the antigen–antibody interactions, the epitopes for antibodies from classes I and II, represented by B1 and F11, are largely overlapped, containing 11–13 residues, primarily located on the ER1 (residues 122–124, 127, 139–142, 157, 166–167) and its adjacent ER4 (residues 511–513) from another p72 monomer (Fig. 1b, c; Supplementary Table S3). In addition to ER1 and ER4 from two adjacent monomers, the class III antibody epitopes, e.g., C9 and H3, include extra residues on the ER3 albeit with significantly decreased residues on the ER1 (Fig. 1d; Supplementary Table S3). By contrast, the G6 antibody targets an epitope contributed from all three monomers (Fig. 1e; Supplementary Fig. S7). Of note, all these four classes of antibodies share overlapping footprints on the ER4, indicative of an antigenic supersite for p72. In contrast to pre-existed antibodies isolated from ASFV-infected swine, antibodies obtained from recombinant p72 ER1-4 immunized mice primarily target ER2 and ER4^11^, indicative of immunogenic differences between natural infections in swine and recombinant peptide immunizations in mice. Nonetheless, ER4 is considered as a major antigenic supersite on p72. Comparative analysis of sequences and structures between class II antibodies revealed decreased interactions in F5 due to the paratope alteration (Y51G) when compared to F11, the latter forming additional hydrogen bonds between Y51 in LCDR2 and R139 in p72, which structurally explains the reduced binding affinity for F5 (Supplementary Fig. S11). Authentic virus neutralization assay revealed all mAbs could partially inhibit ASFV infections, among which the class III antibodies (C9 and H3) had relatively better neutralizing activities and G6 exhibited weak potency due to low occupancy and affinity (Supplementary Fig. S12).

An analysis of the conservation of all the available amino acid sequences of p72 reveals that the epitope on p72 is conserved for the binding sites of these four antibodies (Fig. 1f, g; Supplementary Fig. S13). The different binding modes of the antibodies reveal multiple putative mechanisms of p72-mediated neutralization of ASFV. Since p72 assembles in three forms, pentasymmetron, trisymmetron and zipper, the p72 capsomers adopt different arrangements to allow three distinct assembly patterns: “head-to-back”, “head-to-head”, and “back-to-back”^6^ (Fig. 1h). We superimposed the structures of the p72–antibody complexes on the three forms. We found numerous clashes between antibodies (B1, F11, C9 and H3) on adjacent p72s in trisymmetron and zipper, and also a few clashes in pentasymmetron and zipper. These four antibodies have potentials to cause the dissociation of the capsid as a result of the mechanical forces generated by steric clashes. G6 mediates relatively less clashes, only locating inside the p72 trimer in all three forms, which is consistent with the relatively weak neutralization activity (Fig. 1i). Alternatively, the binding of these antibodies possibly causes effects to interfere with the attachment of putative cellular receptors to viral capsid. Based on these observations, we speculate that the neutralization of AFSV by the p72-directed antibodies probably involves steric occlusion of the putative receptor binding sites or disruption of the integrity of the AFSV capsid (Supplementary Fig. S14). In summary, our studies on antibodies targeting the crucial p72 antigen of AFSV reveal the antigenic supersite that may be involved in virus neutralization and hence provide a framework for the development of vaccines to stall the spread of AFSV.

### Supplementary information

Supplementary Information

## Data Availability

Cryo-EM density maps of the p72-B1, p72-C9, p72-F11, p72-H3 and p72-G6 complexes have been deposited at the Electron Microscopy Data Bank with accession codes EMD-38893, EMD-38894, EMD-38895, EMD-38896 and EMD-38897, and related atomic models have been deposited in the protein data bank under accession codes 8Y3O, 8Y3P, 8Y3Q, 8Y3R and 8ZL9, respectively.
