# Supplementary material for: p72 antigenic mapping reveals a potential supersite of vulnerability for African swine fever virus
Source: Cell Discov. 2024 Jul 31;10:80. doi: 10.1038/s41421-024-00692-x (PMC11289257; doi:10.1038/s41421-024-00692-x)
Supplement: Supplementary file 1 — Supplementary Information [file 41421_2024_692_MOESM1_ESM.pdf]

Supplementary information
Methods and Materials

Ethics statements

This study was carried out in strict accordance with the recommendations in the Guide for the Care and Use of Laboratory Animals of the Ministry of Science and Technology of the People's Republic of China. The protocols were approved by the Committee on the Ethics of Animal Experiments of the Harbin Veterinary Research Institute (HVRI) of the Chinese Academy of Agricultural Sciences (CAAS) and the Animal Ethics Committee of Heilongjiang Province, China.

Biosafety statement and facility

All experiments with live ASF viruses were conducted within the enhanced biosafety level 3 (P3+) and level 4 (P4) facilities in the HVRI of the CAAS approved by the Ministry of Agriculture and Rural Affairs and China National Accreditation Service for Conformity Assessment.

Sample collection

To obtain monoclonal antibodies from ASFV infected swine, we construct antibody library by phage display technology. Briefly, 10mL peripheral blood sample was collected from one ASFV convalescing pig in Yunnan province of China. Peripheral blood mononuclear cells (PBMCs) were isolated by using Ficoll-Paque Plus (GE Healthcare) density gradient media according to the manufacturer’s protocol. Briefly, blood samples were diluted with the same volume of phosphate buffered saline (PBS) (Gibco). The diluted blood was slowly transferred over Ficoll-Paque in SepMate-50 tube (Stemcell). After horizontal centrifugation at room temperature at 800 g for 20 minutes, PBMCs were collected and transferred into a new centrifuge tube. Following two steps of washing with PBS, total RNA of PBMCs was extracted by using RNeasy Mini Kit (Qiagen, Valencia, CA) following the manufacturer’s instructions.

Screening of porcine monoclonal antibodies

Total RNA was used as templates for cDNA synthesis by Transcriptor High Fidelity cDNA Synthesis kit (Roche, Mannheim, Germany). Based on variable genes repertoire listed in IMGT database (www.imgt.org), we designed and synthesized primers specific to porcine variable genes. Variable region of heavy chain, lambda and kappa light chains were amplified by PCR. ScFv antibody library was constructed following a standard protocol. Briefly, recombinant p72 protein was used for panning the ScFv library. Briefly, in the first panning of the antibody library, 30 µg/mL p72 was coated in the immune tubes and incubated at 4°Covernight. After washing by TBST, the phage antibody library pool was added and the tubes were incubated at 37 °C for 2 hours,

followed by removal of the supernatant and washing with TBST for five times. Finally, the enriched phage antibody library pool was eluted using 0.1 M Glycine-HCl (pH 2.2). Using the same procedure, 20 μg/mL and 10 μg/mL of p72 were used respectively for the second and third panning for screening specific binding antibodies pool by phage display. After three rounds of panning, clones were picked and evaluated by phage ELISA. Phage antibodies were got and confirmed by sequencing.

Production of porcine monoclonal antibodies

For recombinant porcine mAb production, we firstly construct expression plasmids containing the porcine IgG1 heavy chain, kappa and lambda light chain constant regions, respectively. Then variable regions of each mAb were cloned into expression vectors. Recombinant mAbs were expressed in 293F cells (Life Technologies, USA) by co-transfection of paired heavy and light chain expression plasmids. Porcine antibodies were purified by Protein-A (GE Healthcare, USA) affinity chromatography were stored at -80 °C for use.

Cell culture, viruses

Primary porcine alveolar macrophages (PAMs) were collected from 30-40-day-old specific-pathogen-free (SPF) pigs, and the cells were maintained in 10% FBS RPMI 1640 medium (Thermo Scientific, USA) at 37°C with 5% CO_2. HLJ/18-6GD was previously generated by deleting six viral genes and replacing them with eGFP (ASFV-eGFP)(Chen et al., 2020).

Virus neutralization test

Spread PAMs onto a 96 well cell culture plate (2.5×10^5 /well). Then mix p72 mAbs with virus solution (MOI=0.1), the final antibody concentrations were 2 mg/mL, 1 mg/mL and 0.5 mg/mL respectively, then incubate the mixture at 37°C with 5% CO_2 for 1 hour. After incubation, discard the cell culture medium from the 96 well cell culture plate and add the above mixture. There are no antibodies and viruses in the positive control; The negative control has neither antibodies nor viruses. Place the culture plate in a 37 °C 5% CO_2 incubator for 48 hours. After 48 hours, capture fluorescence images using a fluorescence microscope.

qPCR

ASFV genomic DNA was extracted from cell supernatants by using GenEluteTM Mammalian Genomic DNA Miniprep Kits (Sigma Aldrich, USA). qPCR was carried out on a QuantStudio 5 system (Applied Biosystems, USA) according to the OIE-recommended procedure as previously described (King et al., 2003).

Production of Fab fragments

The 9 purified monoclonal antibodies were processed to obtain their Fab fragments using the Pierce FAB preparation kit (Thermo Scientific). The samples were first applied to desalination columns to remove the salt and the flow-throughs containing the protein were collected. The de-salinated protein was mixed with beads containing immobilized papain and incubated for 5 h at 37 °C to cleave Fab fragments from the whole antibodies. At the end of incubation time, the reaction mixture was loaded onto a protein A column and the flow-through containing the Fab fragments was collected and dialyzed against PBS (ThermoFisher, catalogue (cat.) no. 10010023).

Cloning, expression and purification

The ASFV genes *B646L* and *B602L* (strain China Pig/HLJ/2018, GenBank: MK333180.1) were cloned into the pCMV vector separately. An N- terminal 10×His-3×Flag tag was added to the recombinant p72 (gene product of B646L) and an N-terminal 1×Strep were added to the recombinant B602L. The transfected HEK293F cells were harvested 48 hours post-transfection by centrifugation at 1000×g for 20 minutes. The cell pellet was resuspended in a buffer containing 20 mM HEPES (pH 7.4), 300 mM NaCl and the suspended cells were sonicated for 3 mins, and then centrifuged to collect the cell lysate supernatant, which was applied to the anti-flag affinity beads to capture the recombinant p72 protein. The eluted sample was concentrated and further purified by a Superdex 200 increase 10/300 GL size exclusion column (GE healthcare) using a buffer containing 20 mM HEPES at pH 7.4 and 300 mM NaCl. All the Fab fragments were generated using a PierceTM Fab Preparation Kit (Thermo Fisher Scientific) according to the manufacturer′s instructions.

Surface plasmon resonance

p72 trimer was immobilized onto a CM5 sensor chip surface using the NHS/EDC method using Biacore 8k (GE Healthcare) and PBS as running buffer (with 0.05% Tween-20). B1, H5, D7, E5, F11, F5, C9, H3 and G6 were purified for the single-cycle kinetic assays to determine the affinities. The competitive binding assays for antibodies were performed according to the saturation concentration. The first antibody flew over the chip at a rate of 10 μL/min. After saturation with the first antibody, the other antibodies individually were injected at the same rate for another 120 s. The data were analyzed using Biacore 8k Evaluation Software (GE Healthcare).

Cryo-EM sample preparation, data acquisition and structure determination

Purified p72 and five Fabs were mixed in a molar ratio of 1:1.3 respectively to a final concentration of 0.8 mg/mL on ice. Immediately after this, 3.5 μL of the complex was deposited on Cu-300 1.2/1.3 grids (QUANTIFOIL) that had been freshly glow-discharged in a Solarus 950 plasma cleaner (Gatan). The excess liquid was blotted for 3 s with a force of 2 using a Vitrobot (Thermo Fisher Scientific) and plunged into liquid ethane.

The Cryo-EM datasets of p72 in complex with B1 were collected at 300 kV using Titan Krios microscope (Thermo Fisher Scientific) equipped with a K2 detector (Gatan, Pleasanton, CA). Movies (32 frames, defocus of –1.2 to –1.8 μm, total dose of 60 e^{-} $Å^{-2}$) were recorded using SerialEM yielding the final pixel size of 1.04 Å. The images of p72 in complex with C9 were collected by Arctica microscope equipped with a K2 detector (Gatan) at 300 kV and movies (32 frames, defocus of –1.2 to –1.8 μm, total dose of 60 e^{-} $Å^{-2}$) were collected by SerialEM with a pixel size of 1.0 Å. The Cryo-EM datasets of p72 in complex with F11, H3 and G6 were collected at 300 kV using Titan Krios microscope (Thermo Fisher Scientific) equipped with a K3 detector (Gatan, Pleasanton, CA). Movies (32 frames, defocus of –1.2 to –1.8 μm, total dose of 60 e^{-} $Å^{-2}$) were recorded using SerialEM resulting in the final pixel size of 1.07 Å. A full description of the data collection parameters can be found in Table S2.

Cryo-EM data processing

A total of 3,550, 6,360, 3,242, 4,697 and 5,831 micrographs of p72 in complex with B1, F11, C9, H3 and G6, respectively were recorded and subjected to beam-induced motion correction using motionCorr in Relion 3.0 package. The defocus value of each image was calculated by Gctf. Then, 1,724,905, 4,285,966, 642,943, 1,814,761 and 3,650,050 particles of the p72 in complex with B1, F11, C9, H3 and G6, respectively, were picked and extracted for reference-free 2D alignment by cryoSPARC, based of which, 210,044, 531,306, 223,575, 296,696 and 265,660 particles were selected and applied for 3D classification by Relion3.0 for p72 in complex with B1, F11, C9, H3 and G6, respectively with C3 symmetry imposed to produce the potential conformations for the complexes except G6 with C1. Afterwards, the candidate model for each complex was selected and processed by auto-refinement and postprocessing in cryoSPARC to generate the final cryo-EM density for p72 in complex with B1, F11, C9, H3 and G6. Local refinement was performed to further improve the resolution of the binding interface of these five complexes. All the resolutions were evaluated on the basis of the gold-standard Fourier shell correlation (threshold = 0.143). The

local resolution was evaluated by ResMap. All dataset processing is shown in Fig. S3 and summarized in Table S2.

Model fitting and refinement

The atomic models of the complexes were generated by first fitting the chains of the native apo p72 trimer (PDB number of 6KU9) and Fabs (PDB number of 3UX9 and 5A3I for B1, 3UX9 and 4O9H for F11, C9 as well as for H3) into the cryo-EM densities of the final p72-Fab-complexes described above by Chimera, followed by manually adjustment and correction according to the protein sequences and densities in Coot, as well as real space refinement using Phenix. Details of the refinement statistics of the complexes are summarized in Table S2.

Supplementary Figures

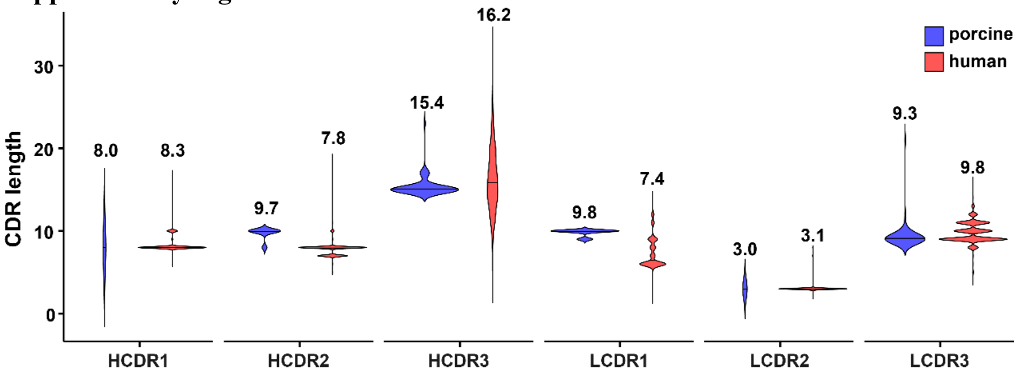

Supplementary information, Fig. S1 CDR length comparison between human and porcine. The blue color represents the porcine CDR length. The red color represents human's CDR length. 59 porcine antibodies and 8,197 human antibodies were used for above analysis.

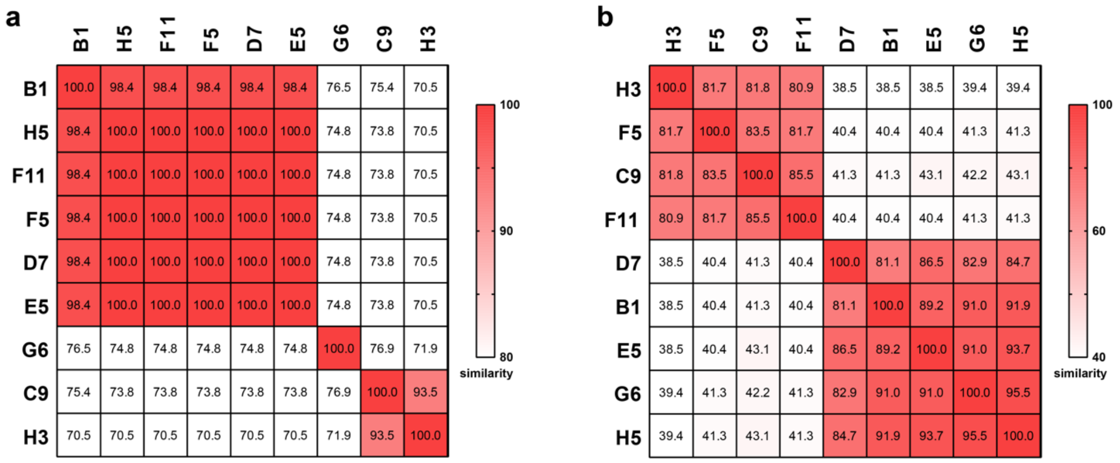

Supplementary information, Fig. S2 Cluster analysis of the sequence comparison of the (a) heavy chain and (b) light chain of the antibodies. The number in each box represent the identity between two antibodies.

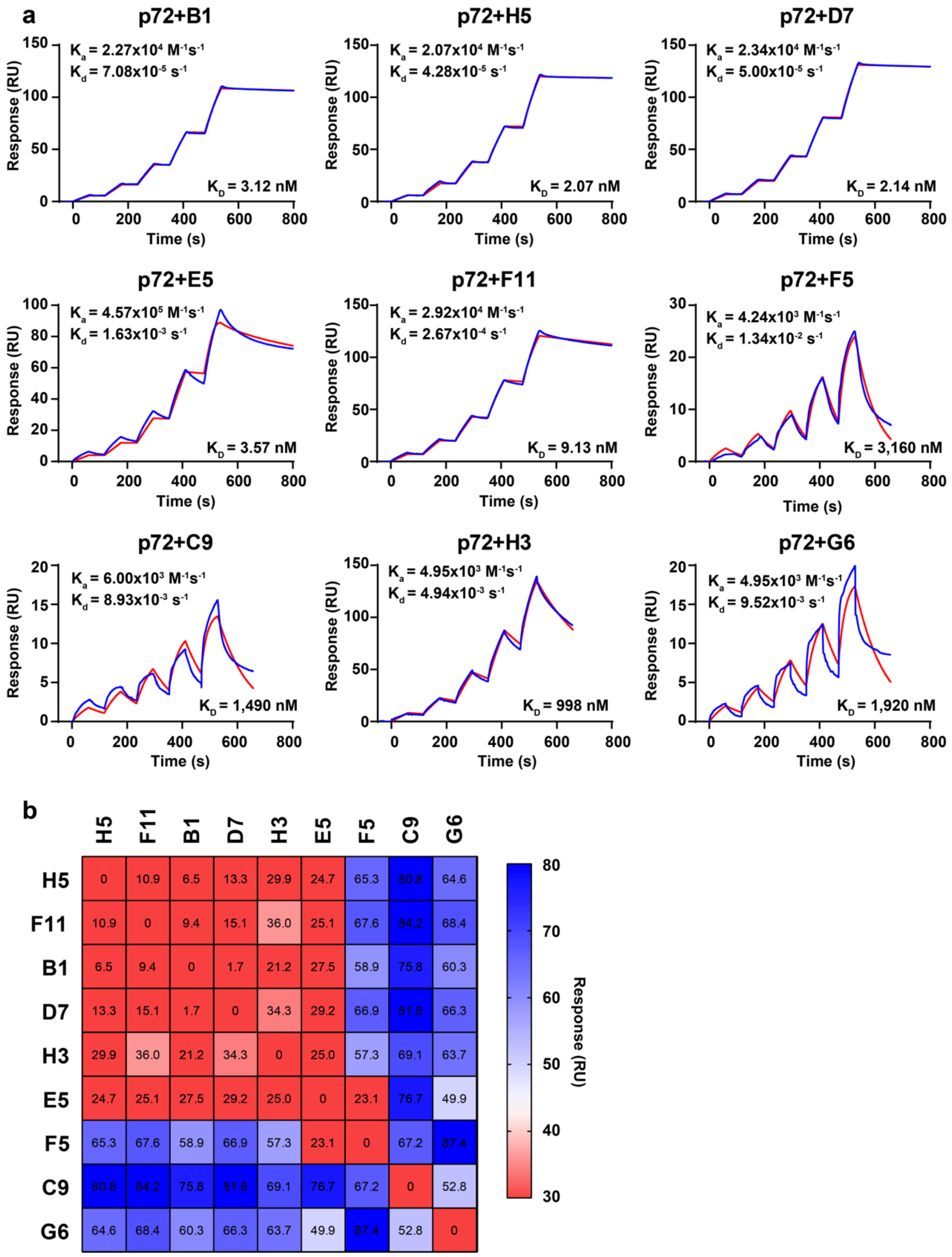

Supplementary information, Fig. S3 The binding affinities of B1, H5, D7, E5, F11, F5, C9, H3 and G6 to p72. (a) Binding curves of B1, H5, D7, E5, F11, F5, H3, C9, and G6 against p72 were measured by SPR. (b) Results of clustering of antibodies based on antibody competition assays by SPR. The competitive binding signal values are colored from red (low values) to blue (high values).

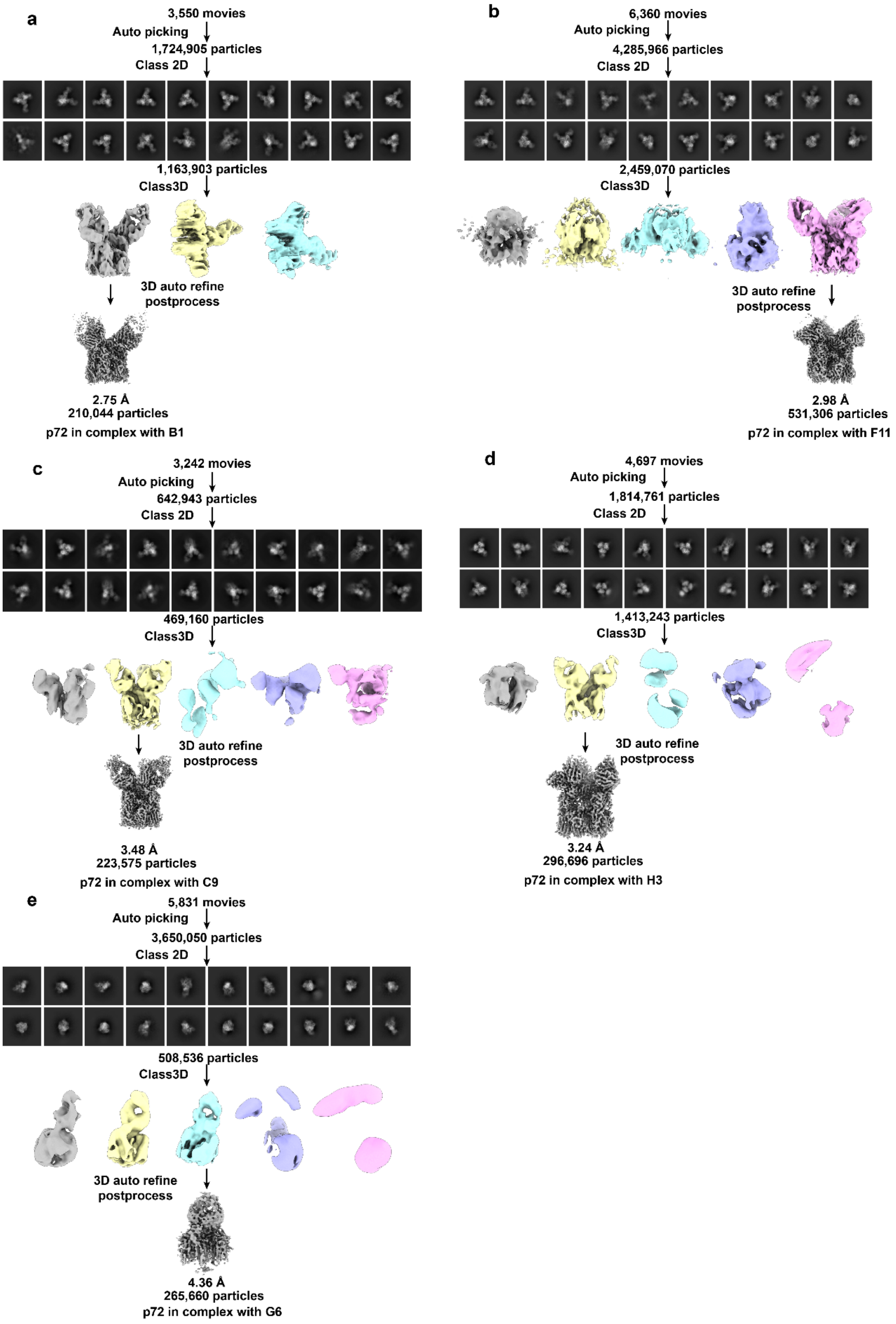

Supplementary information, Fig. S4 Flowcharts for data processing. Flowcharts for p72 trimer in complex with (a) B1, (b) F11, (c) C9, (d) H3 and (e) G6.

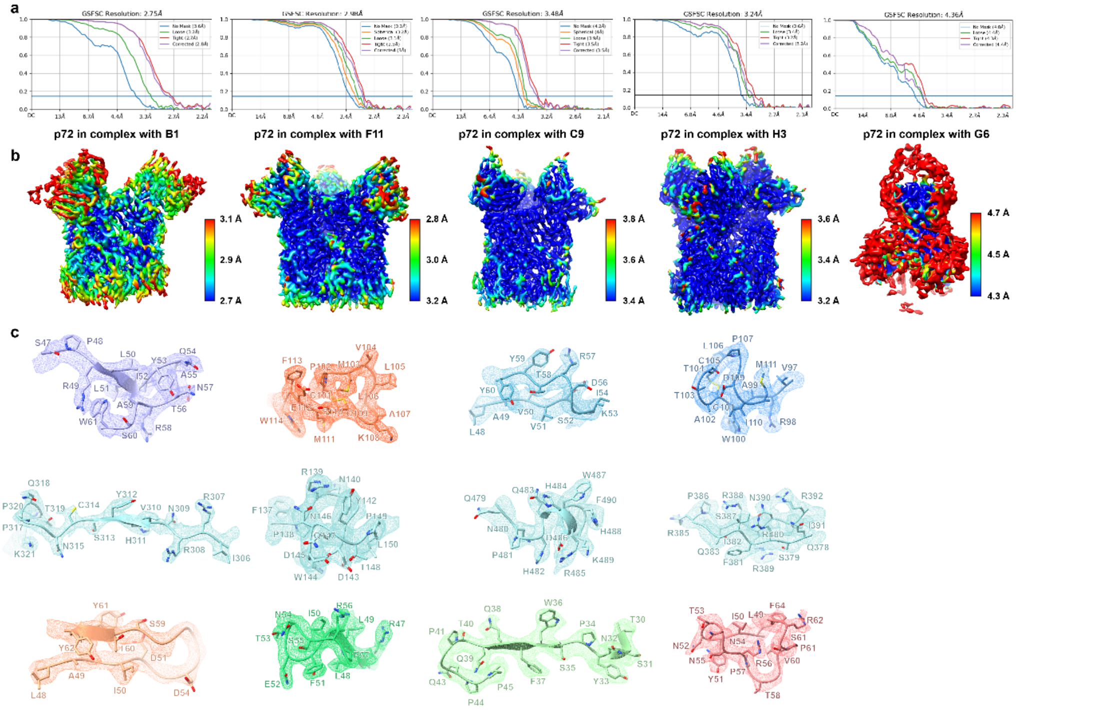

Supplementary information, Fig. S5 Resolution estimation of the EM maps. (a) The gold standard FSC curves of overall maps of p72 trimer in complex with Fab B1, F11, C9, H3 and G6. (b) Local resolution assessments of cryo-EM maps using ResMap are shown. (c) Cryo-EM density maps of p72 in complex with B1, F11, C9 and H3 and their interfaces are shown. Color scheme is the same as in Fig.1. Residues are shown as sticks with oxygen colored in red, nitrogen colored in blue and sulfurs colored in yellow, respectively.

a

	PDB	antibody name	Organism	RMSD
Heavy chain of B1	3UX9	AIFNα1bIgG01	Homo sapiens	0.777
Light chain of B1	5A3I	FLD194	Homo sapiens	0.730
Heavy chain of F11	3UX9	AIFNα1bIgG01	Homo sapiens	0.794
Light chain of F11	4O9H	61H7	Lama glama	0.587
Heavy chain of C9	3UX9	AIFNα1bIgG01	Homo sapiens	0.793
Light chain of C9	4O9H	61H7	Lama glama	0.616
Heavy chain of H3	3UX9	AIFNα1bIgG01	Homo sapiens	0.837
Light chain of H3	4O9H	61H7	Lama glama	0.828

b

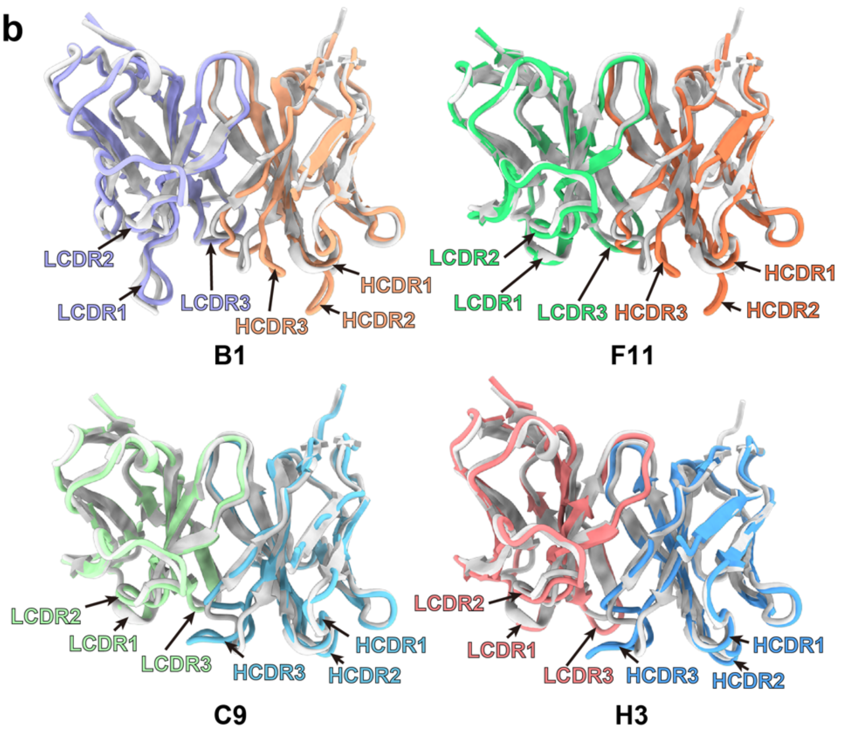

Supplementary information, Fig. S6 Information on the best matching antibodies in the PDB database. (a) The RMSD of chains of antibodies with their most similar protein in PDB. (b) The structure comparison of each chain of antibodies with their most similar protein in PDB. Color scheme is the same as in Fig.1. Human antibodies are colored by grey.

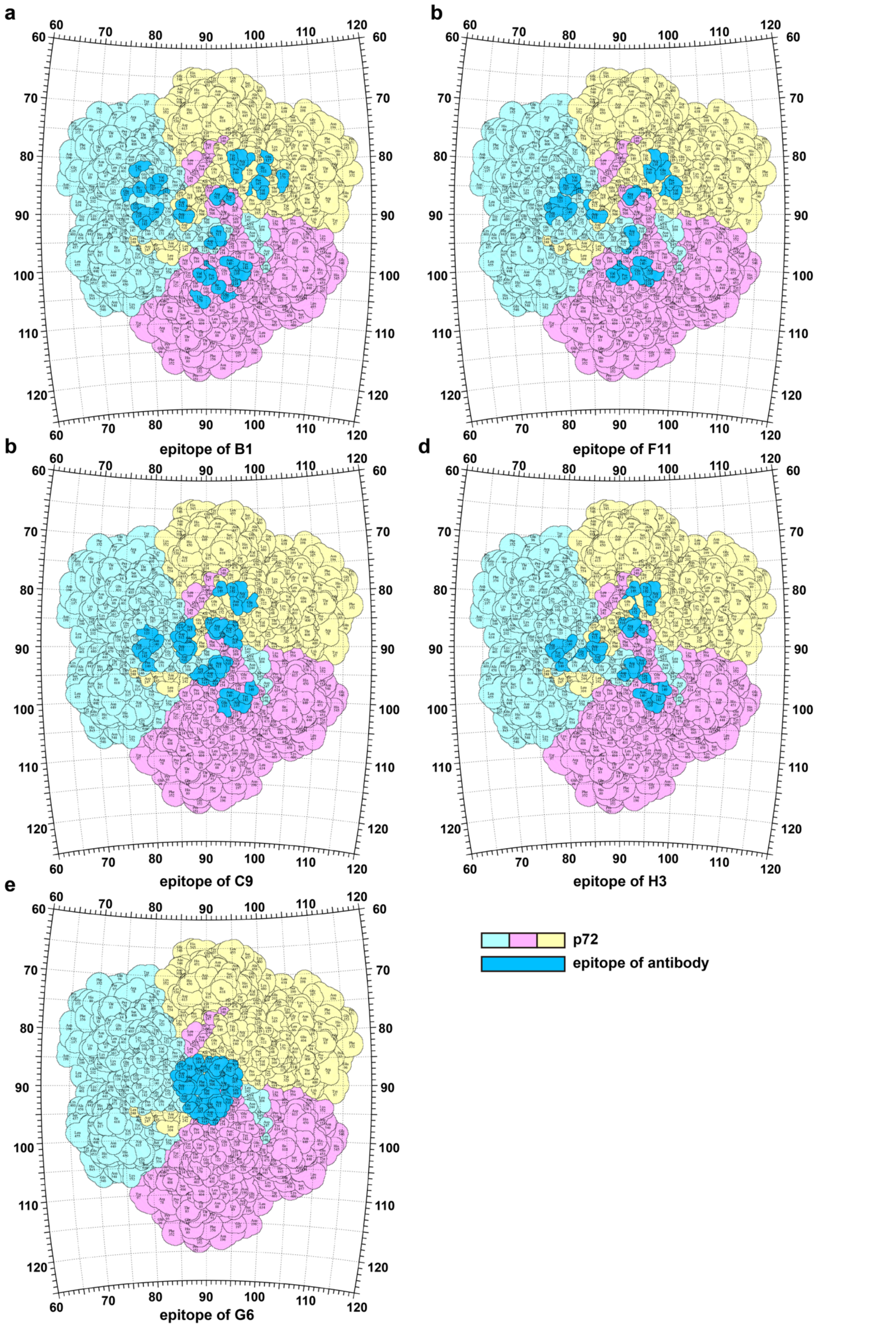

Supplementary information, Fig. S7 The footprint of the p72 surface. A two-dimensional projection of the p72 surface was produced using RIVEM. The epitopes of B1(a), F11(b), C9(c), H3(d) and G6(e) are colored with dark blue.

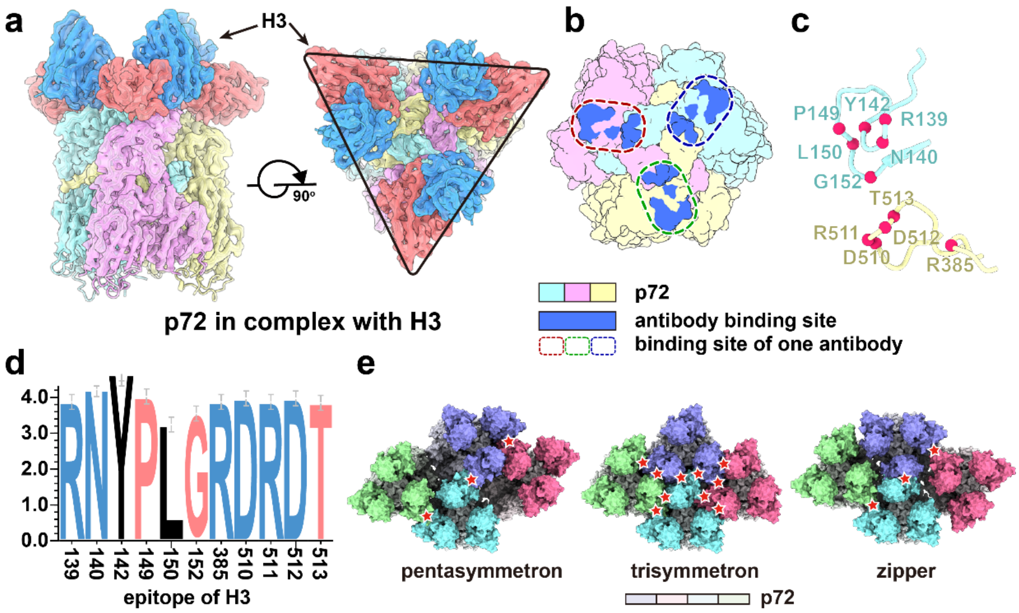

Supplementary information, Fig. S8 Structural imformation of p72 in complex with H3. (a) Side and top views of Cryo-EM maps of p72 in complex with H3. (b) Interaction region between p72 and H3 on p72. Dark blue areas display antibody binding site. The dashed box represents the binding site of one antibody. (c) The enlarged panel show residues involved in the interaction with antibodies. (d) Sequence conservation analysis of the epitopes of H3. (e) Possible locations of antibody binding to different assemblies of p72. The clashes between the antibodies are marked with a red star. The bar at the bottom shows the color of p72.

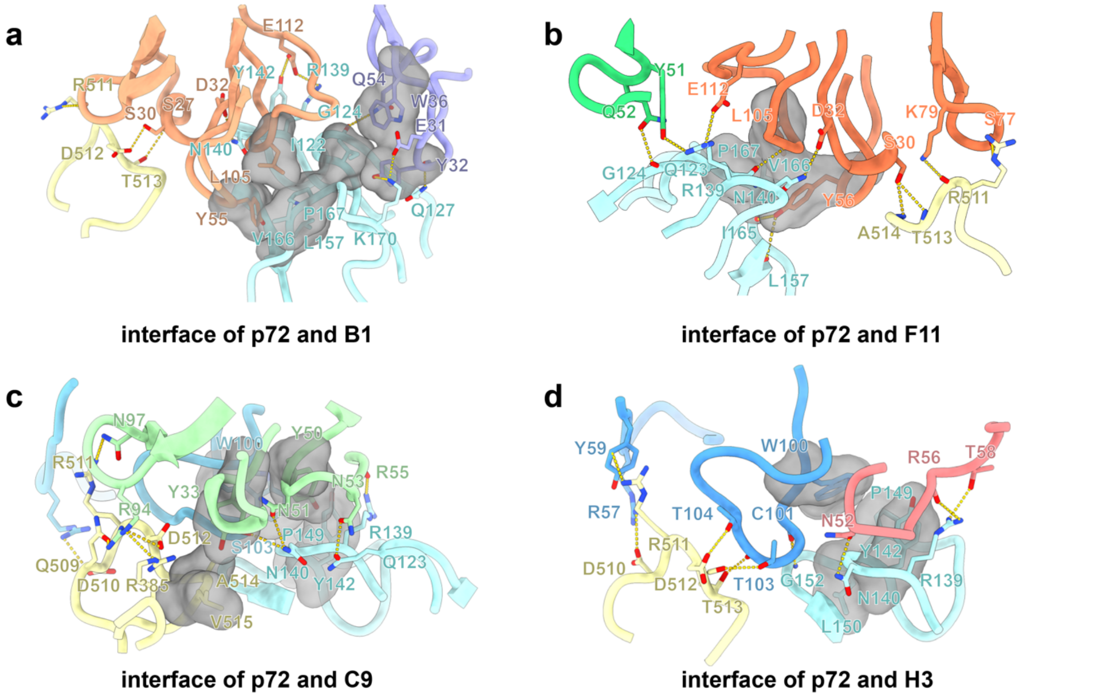

Supplementary information, Fig. S9 Details of Structural comparison of the interface of the complex with B1(a), F11(b), C9(c) and H3(d). The yellow dashed lines represent hydrophilic interactions and the gray transparent surfaces represent hydrophobic pockets formed by hydrophobic interactions.

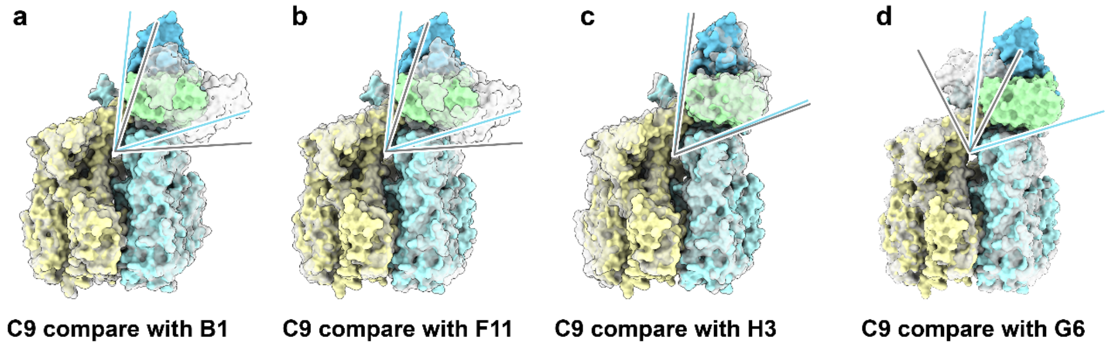

Supplementary information, Fig. S10 Comparison of binding modes of different antibodies. All structures are displayed in the same orientation. Angles formed between two different antibodies during their binding with p72 are marked out.

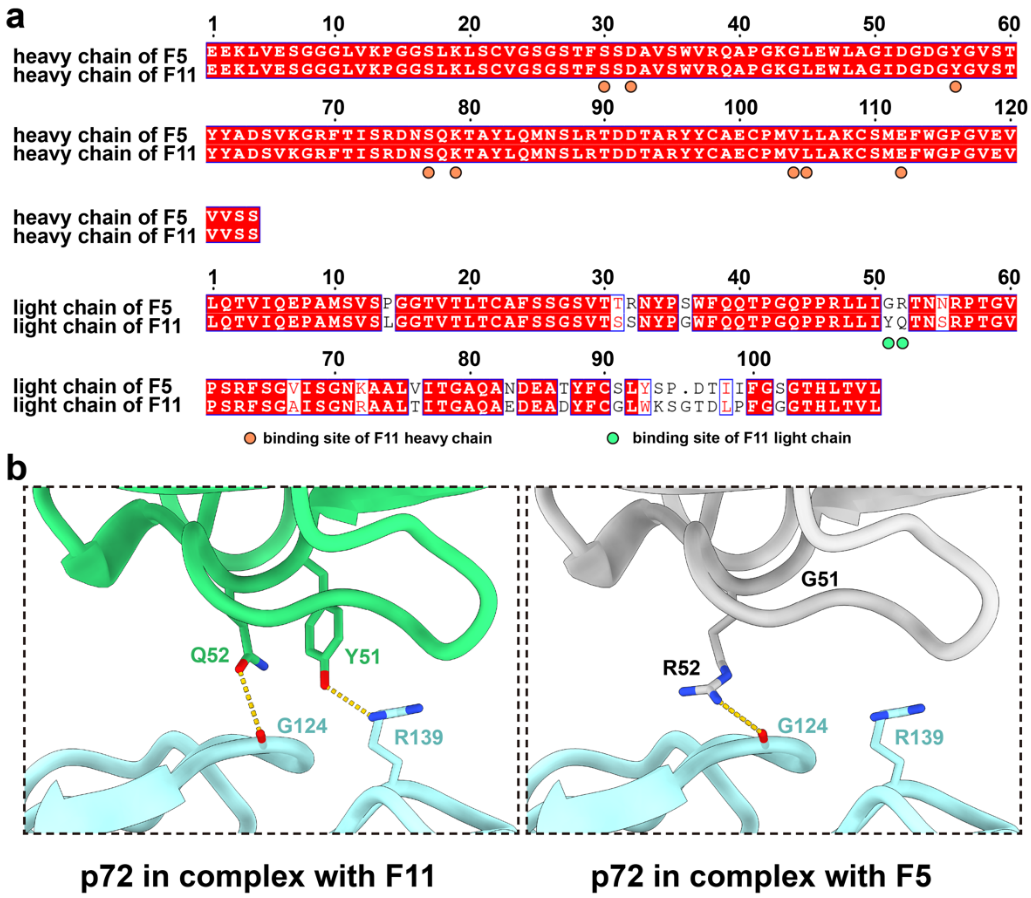

Supplementary information, Fig. S11 Comparison of F11 and F5. (a) Multiple sequence alignment results of F5 and F11. The paratope resides in heavy and light chains are marked by orange and green circles, respectively. (b) Binding mode of F11 and F5 in LCDR2. The yellow dashed lines represent hydrophilic interactions.

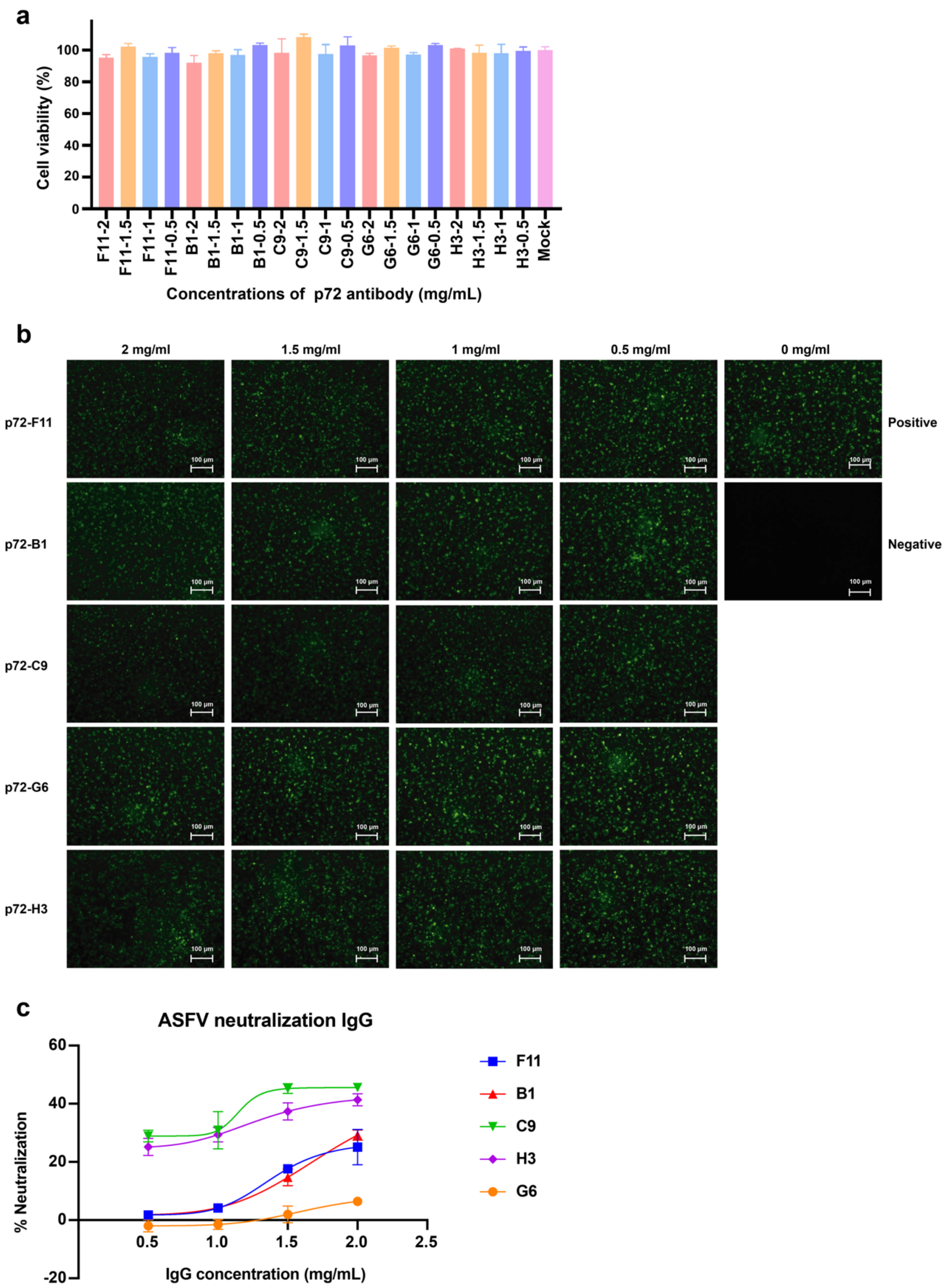

Supplementary information, Fig. S12 ASFV replication neutralized by p72 monoclonal antibody in PAMs. (a) Cell viability detection at experimental concentrations of monoclonal antibodies. (b) eGPF fluorescence indicated ASFV-infected cells using fluorescence microscopy. Scale bar indicated 100 μm. (c) ASFV p72 gene levels were analyzed by qPCR. Each value represents the average of three independent experiments.

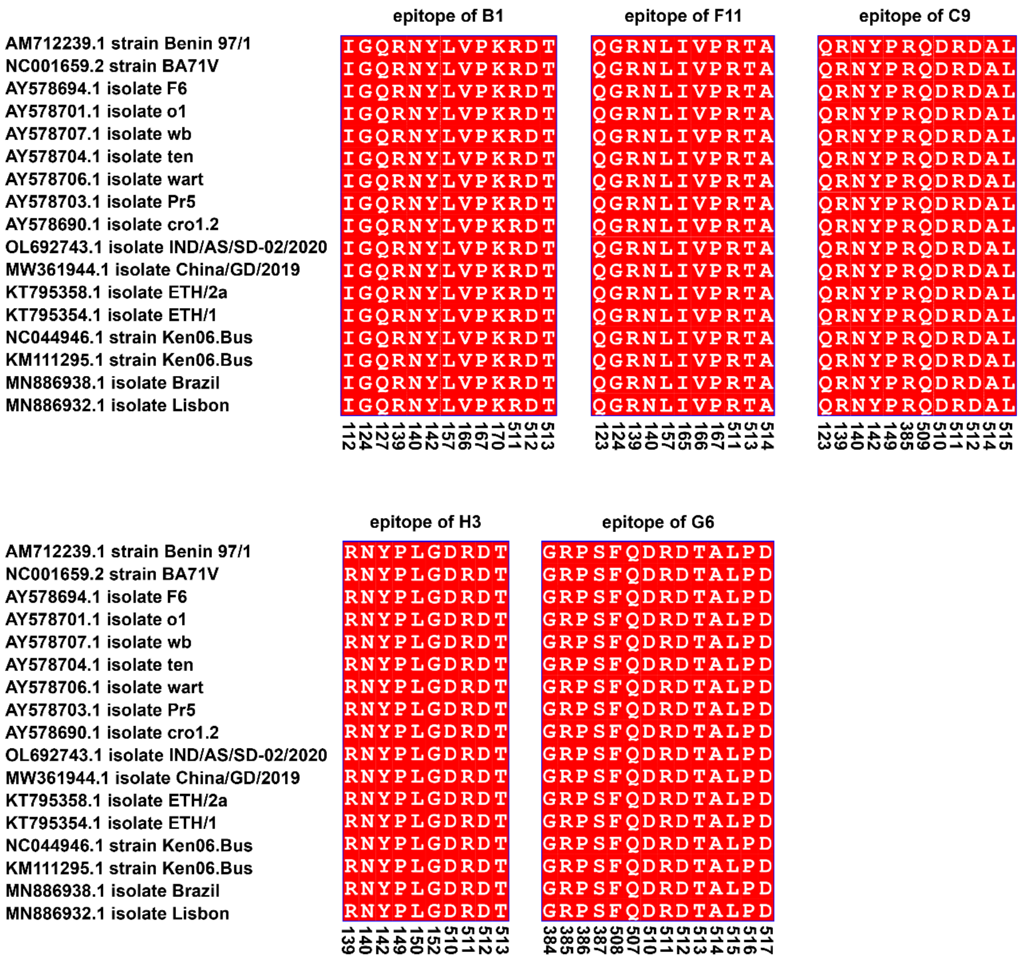

Supplementary information, Fig. S13 Sequence alignment analysis. Comparison of sequence conservation in 17 ASFV strains. Regions involved in the interaction with B1(left), F11(middle), C9(right), H3(lower left) and G6(lower right) are shown.

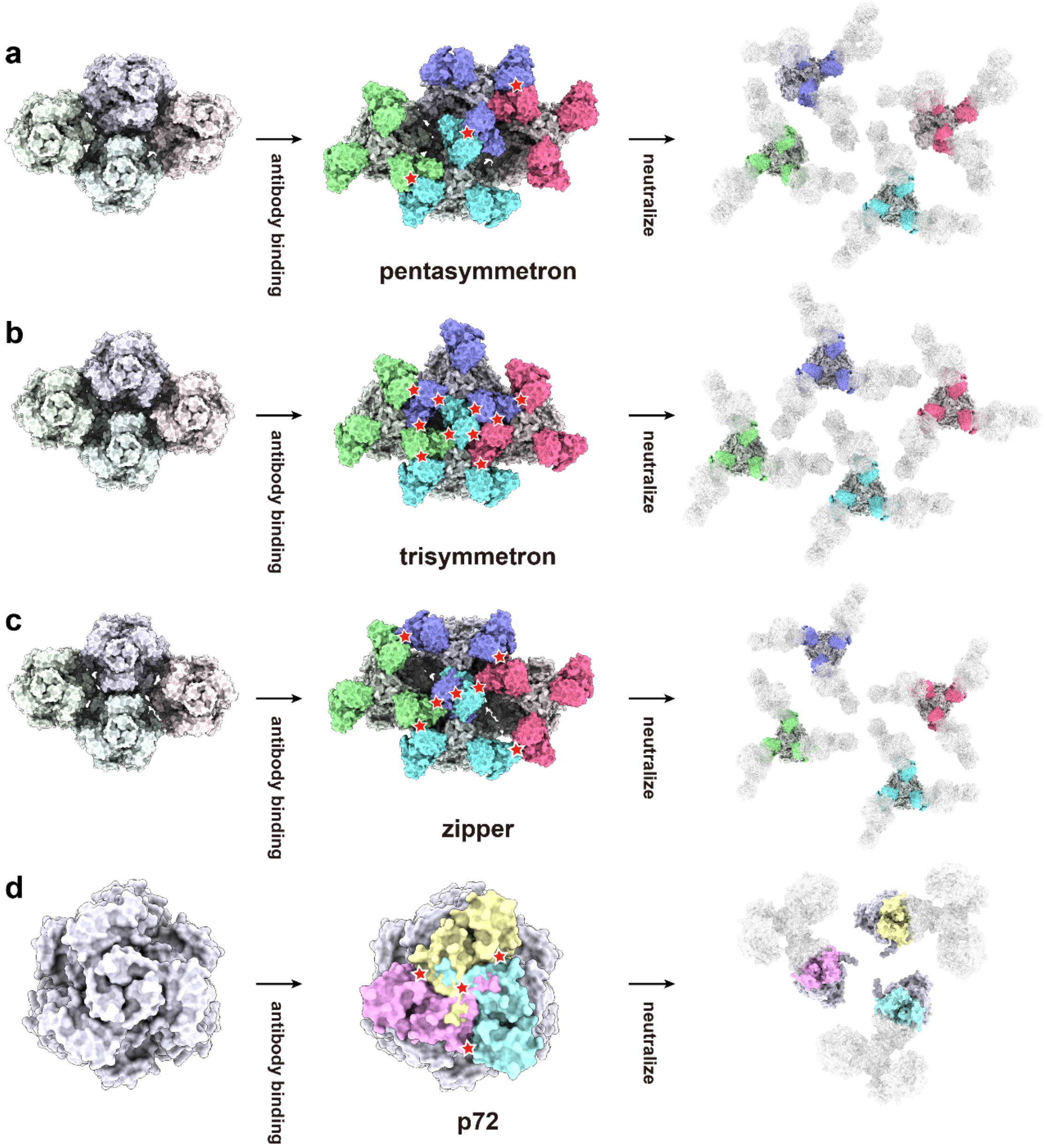

Supplementary information, Fig. S14 Neutralizing mode of different antibody binding site. Possible locations of antibody binding in different organizations. The clash between antibodies was marked with red star.

Supplementary information, Table S1 | The complementary determining region (CDR) residues and length of 59 antibodies.

antibody name	HCDR1	length	HCDR2	length	HCDR3	length	LCDR1	length	LCDR2	length	LCDR3	length
H11	GFTFSS<br>YE	8	VDDG<br>DWSA<br>ED	10	AIGGVFDYS<br>GCYSGPPG<br>YYAMDL	23	SGSVT<br>TSNY	9	QTN	3	ALGKS<br>CTGI	9
C12	GFTFSS<br>YE	8	VSKIG<br>DRT	8	VRAWCAST<br>CLPGDIMDL	17	SGSVT<br>SSSL	9	NTN	3	ALSKG<br>SGTYS<br>YI	12
C1	GFTFSS<br>YE	8	VSKIG<br>DRT	8	VRAWCAST<br>CLPGDIMDL	17	SGSVT<br>SSSL	9	NTN	3	ALSKG<br>SGTYS<br>YIFGGG<br>TRLSV	21
E12	GFTFSS<br>YE	8	VSKIG<br>DRT	8	VRAWCAST<br>CLPGDIMDL	17	SGSVT<br>TSNY	9	STN	3	ALYKS<br>GVIV	9
B1	GSTFSS<br>DA	8	IDGDG<br>YGVST	10	AECPMVLL<br>AKCSMEF	15	QSLEE<br>YGKN<br>W	10	QAT	3	FQDLQ<br>PPNG	9
B2	GSTFSS<br>DA	8	IDGDG<br>YGVST	10	AECPMVLL<br>AKCSMEF	15	QSLEE<br>YGKN<br>W	10	QAT	3	FQDLQ<br>PPNG	9
A9	GSTFSS<br>DA	8	IDGDG<br>YGVST	10	AECPMVLL<br>AKCSMEF	15	QSLEE<br>YGKN<br>W	10	QAT	3	FQDLQ<br>PPNG	9
C3	GSTFSS<br>DA	8	IDGDG<br>YGVST	10	AECPMVLL<br>AKCSMEF	15	QSLEE<br>YGKN<br>W	10	QAT	3	FQDLQ<br>PPNG	9
C5	GSTFSS<br>DA	8	IDGDG<br>YGVST	10	AECPMVLL<br>AKCSMEF	15	QSLEE<br>YGKN<br>W	10	QAT	3	FQDLQ<br>PPNG	9
G1	GSTFSS<br>DA	8	IDGDG<br>YGVST	10	AECPMVLL<br>AKCSMEF	15	QSLEE<br>YGKN<br>W	10	QAT	3	FQDLQ<br>PPNG	9
E4	GSTFSS<br>DA	8	IDGDG<br>YGVST	10	AECPMVLL<br>AKCSMEF	15	QSLEE<br>YGKN<br>W	10	QAT	3	FQYLQ<br>FPNG	9
F11	GSTFSS<br>DA	8	IDGDG<br>YGVST	10	AECPMVLL<br>AKCSMEF	15	SGSVT<br>SSNY	9	QTN	3	GLWKS<br>GTDLP	10
E7	GSTFIS<br>DA	8	IDGDG<br>YGVST	10	AECPMVLL<br>AKCSMEF	15	SGSITN<br>NNY	9	QTN	3	GLWKS<br>GTDLP	10
C9	GFTFSS<br>YE	8	VSKIG<br>DRT	8	VRAWCAST<br>CLPGDIMDL	17	SGSVT<br>TSNY	9	NTN	3	GLYKR<br>SANNI	10
G12	GFTFSS<br>YE	8	VSKIG<br>DRT	8	VRAWCAST<br>CLPGDIMDL	17	SGSVT<br>TSNY	9	NTN	3	GLYKR<br>SANNI	10
F4	GFTFSS<br>YE	8	VSKIG<br>DRT	8	VRAWCAST<br>CLPGDIMDL	17	SGSVT<br>TSNY	9	NTN	3	GLYKR<br>SANNI	10
D7	GSTFSS<br>DA	8	IDGDG<br>YGVST	10	AECPMVLL<br>AKCSMEF	15	QSLEN<br>NRYNF	10	FAT	3	LQNKE<br>YLPG	9
A4	GSTFSS<br>DA	8	IDGDG<br>YGVST	10	AECPMVLL<br>AKCSMEF	15	QSLEN<br>NRYNF	10	FAT	3	LQNKE<br>YLPG	9
D5	GSTFSS<br>DA	8	IDGDG<br>YGVST	10	AECPMVLL<br>AKCSMEF	15	QSLEN<br>NRYNF	10	FAT	3	LQNKE<br>YLPG	9
E5	GSTFSS<br>DA	8	IDGDG<br>YGVST	10	AECPMVLL<br>AKCSMEF	15	QSLEE<br>YGKN<br>W	10	EAT	3	QQQKE<br>SPNG	9
A10	GSTFSS<br>DA	8	IDGDG<br>YGVST	10	AECPMVLL<br>AKCSMEF	15	QSLEE<br>YGKN<br>W	10	EAT	3	QQQKE<br>SPNG	9
B10	GSTFSS<br>DA	8	IDGDG<br>YGVST	10	AECPMVLL<br>AKCSMEF	15	QSLEE<br>YGKN<br>W	10	EAT	3	QQQKE<br>SPNG	9
H5	GSTFSS<br>DA	8	IDGDG<br>YGVST	10	AECPMVLL<br>AKCSMEF	15	QSLEE<br>YGKN<br>W	10	QAT	3	QQYKE<br>FPNG	9
C8	GSTFSS<br>DA	8	IDGDG<br>YGVST	10	AECPMVLL<br>AKCSMEF	15	QSLEE<br>YGKN<br>W	10	QAT	3	QQYKE<br>FPNG	9
E6	GSTFSS<br>DA	8	IDGDG<br>YGVST	10	AECPMVLL<br>AKCSMEF	15	QSLEE<br>YGKN<br>W	10	QAT	3	QQYKE<br>FPNG	9
C10	GSTFSS<br>DA	8	IDGDG<br>YGVST	10	AECPMVLL<br>AKCSMEF	15	QSLEE<br>YGKN<br>W	10	QAT	3	QQYKE<br>FPNG	9
H4	GSTFSS<br>DA	8	IDGDG<br>YGVST	10	AECPMVLL<br>AKCSMEF	15	QSLEE<br>YGKN<br>W	10	QAT	3	QQYKE<br>FPNG	9

D11	GSTFSSDA	8	IDGDGYGVST	10	AECPMVLLAKCSMEF	15	QSLEEYGKNW	10	QAT	3	QQYKEFPNG	9
E8	GSTFSSDA	8	IDGDGYGVST	10	AECPMVLLAKCSMEF	15	QSLEEYGKNW	10	QAT	3	QQYKEFPNG	9
F6	GSTFSSDA	8	IDGDGYGVST	10	AECPMVLLAKCSMEF	15	QSLEEYGKNW	10	QAT	3	QQYKEFPNG	9
F9	GSTFSSDA	8	IDGDGYGVST	10	AECPMVLLAKCSMEF	15	QSLEEYGKNW	10	QAT	3	QQYKEFPNG	9
C6	GSTFSSDA	8	IDGDGYGVST	10	AECPMVLLAKCSMEF	15	QSLEEYGKNW	10	QAT	3	QQYKEFPNG	9
E10	GSTFSSDA	8	IDGDGYGVST	10	AECPMVLLAKCSMEF	15	QSLEEYGKNW	10	QAT	3	QQYKEFPNG	9
D4	GSTFSSDA	8	IDGDGYGVST	10	AECPMVLLAKCSMEF	15	QSLEEYGKNW	10	QAT	3	QQYKEFPNG	9
H12	GSTFSSDA	8	IDGDGYGVST	10	AECPMVLLAKCSMEF	15	QSLEEYGKNW	10	QAT	3	QQYKEFPNG	9
A5	GSTFSSDA	8	IDGDGYGVST	10	AECPMVLLAKCSMEF	15	QSLEEYGKNW	10	QAT	3	QQYKEFPNG	9
A2	GSTFSSDA	8	IDGDGYGVST	10	AECPMVLLAKCSMEF	15	QSLEEYGKNW	10	QAT	3	QQYKEFPNG	9
B3	GSTFSSDA	8	IDGDGYGVST	10	AECPMVLLAKCSMEF	15	QSLEEYGKNW	10	QAT	3	QQYKEFPNG	9
G9	GSTFSSDA	8	IDGDGYGVST	10	AECPMVLLAKCSMEF	15	QSLEEYGKNW	10	QAT	3	QQYKEFPNG	9
H1	GSTFSSDA	8	IDGDGYGVST	10	AECPMVLLAKCSMEF	15	QSLEEYGKNW	10	QAT	3	QQYKEFPNG	9
B7	GSTFSSDA	8	IDGDGYGVST	10	AECPMVLLAKCSMEF	15	QSLEEYGKNW	10	QAT	3	QQYKEFPNG	9
G11	GSTFSSDA	8	IDGDGYGVST	10	AECPMVLLAKCSMEF	15	QSLEEYGKNW	10	QAT	3	QQYKEFPNG	9
D12	GSTFSSDA	8	IDGDGYGVST	10	AECPMVLLAKCSMEF	15	QSLEEYGKNW	10	QAT	3	QQYKEFPNG	9
E1	GSTFSSDA	8	IDGDGYGVST	10	AECPMVLLAKCSMEF	15	QSLEEYGKNW	10	QAT	3	QQYKEFPNG	9
D9	GSTFSSDA	8	IDGDGYGVST	10	AECPMVLLAKCSMEF	15	QSLEEYGKNW	10	QAT	3	QQYKEFPNG	9
D6	GSTFSSDA	8	IDGDGYGVST	10	AECPMVLLAKCSMEF	15	QSLEEYGKNW	10	QAT	3	QQYKEFPNG	9
G4	GSTFSSDA	8	IDGDGYGVST	10	AECPMVLLAKCSMEF	15	QSLEEYGKNW	10	QAT	3	QQYKEFPNG	9
B6	GSTFSSDA	8	IDGDGYGVST	10	AECPMVLLAKCSMEF	15	QSLEEYGKNW	10	QAT	3	QQYKEFPNG	9
G2	GSTFSSDA	8	IDGDGYGVST	10	AECPMVLLAKCSMEF	15	QSLEEYGKNW	10	QAT	3	QQYKEFPNG	9
G10	GSTFSSDA	8	IDGDGYGVST	10	AECPMVLLAKCSMEF	15	QSLEEYGKNW	10	QAT	3	QQYKEFPNG	9
F10	GSTFSSDA	8	IDGDGYGVST	10	AECPMVLLAKCSMEF	15	QSLEEYGKNW	10	QAT	3	QQYKEFPNG	9
C4	GSTFSSDA	8	IDGDGYGVST	10	AECPMVLLAKCSMEF	15	QSLEEYGKNW	10	QAT	3	QQYKEFPNG	9
H2	GSTFSSDA	8	IDGDGYGVST	10	AECPMVLLAKCSMEF	15	QSLEEYGKNW	10	QAT	3	QQYKEFPNG	9
H6	GSTFSSDA	8	IDGDGYGVST	10	AECPMVLLAKCSMEF	15	QSLEEYGKNW	10	QAT	3	QQYKEFPNG	9
G6	GYTFSSYP	8	IDSSGSKM	8	ARGRWCYGVICDMDL	15	QSLEEYGKNW	10	QAT	3	QQYKEFPWT	9
F1	GSTFSSDA	8	IDGDGYGVST	10	AECPMVLLAKCSMEF	15	QSLEEYGKNW	10	QAT	3	QQYKKFPNG	9
A7	GSTFSSDA	8	IDGDGYGVST	10	AECPMVLLAKCSMEF	15	QSLEEYGKNW	10	QAT	3	QQYKKFPNG	9

F5	GSTFSS DA	8	IDGDG YGVST	10	AECPMVLL AKCSMEF	15	SGSVT TRNY	9	RTN	3	SLYSPD TII	9
H3	GFTFIT YE	8	VSKIG DRT	8	VRAWCATT CLPGDIMDL	17	SGSITN NNY	9	NTN	3	TLYISIA DVI	10

Supplementary information, Table S2 | Statistics for cryo-EM data collection, refinement, and validation

	p72 in complex with B1	p72 in complex with F11	p72 in complex with C9	p72 in complex with H3	p72 in complex with G6
**Data collection**					
Voltage (kV)	300	300	200	300	300
Microscope	FEI Titan Krios	FEI Titan Krios	FEI Arctica	FEI Titan Krios	FEI Titan Krios
Camera	K2 (Gatan)	K3 (Gatan)	K2 (Gatan)	K3 (Gatan)	K3 (Gatan)
Magnification (calibrated)	81,000X	81,000X	81,000X	81,000X	81,000X
Electron exposure ($e^{–}/Å^2$)	60	60	60	60	60
Exposure rate ($e^{–}/Å^2/s$)	16.02	16.02	16.02	16.02	16.02
Number of frames collected per micrograph	32	32	32	32	32
Automation software	SerialEM	SerialEM	SerialEM	SerialEM	SerialEM
Defocus range (µm)	–1.2 to –1.8	–1.2 to –1.8	–1.2 to –1.8	–1.2 to –1.8	–1.2 to –1.8
Pixel size (Å)	1.04	1.07	1.00	1.07	1.07
**Overall map processing**					
Micrographs used	3,550	6,360	3,242	4,697	5,831
Symmetry imposed	C3	C3	C3	C3	C1
Initial particle images	1,724,905	4,285,966	642,943	1,814,761	3,650,050
Final particle images	210,044	531,306	223,575	296,696	265,660
Resolution at 0.143 FSC of masked reconstruction (Å)	2.75	2.98	3.48	3.24	4.36
Map sharpening B factor ($Å^2$)	-114.4	-159.7	-193.1	-157.5	-273.8
**Local map refinement**					
Refinement package	Phenix v1.19	Phenix v1.19	Phenix v1.19	Phenix v1.19	
Model composition					
Non-hydrogen atoms	17,115	15,738	17,028	15,735	
Protein residues	2,166	2,016	2,163	2,001	
R.m.s. deviations					
Bond lengths (Å)	0.003	0.003	0.004	0.002	
Bond angles (°)	0.727	0.547	0.77	0.565	
B factors ($Å^2$)					
Protein	68.68	51.54	36.45	82.22	
Validation					
MolProbity score	1.66	1.45	1.71	1.74	
Clashscore	6.14	4.69	6.57	7.97	
Poor rotamers (%)	0	0	0	0	
Ramachandran plot					
Favored (%)	95.4	96.67	95.2	95.62	
Allowed (%)	4.6	3.33	4.8	4.38	
Disallowed (%)	0	0	0	0	

Cb outliers (%)	0	0	0	0
CaBLAM outliers (%)	3.87	1.65	3.97	3.01

Supplementary information, Table S3 | Residues of antibodies Fab fragment interacting with p72 (d < 4 Å).

Complex	p72	Heavy chain		Light chain	
**p72 in complex with B1**	I122			W36	Y32
	G124			Q54	
	Q127			Y32	
	R139	E112			
	N140	D32			
	Y142	E112			
	L157	Y56			
	V166	L105	Y56		
	P167	L105	Y56		
	K170			Y32	E31
	R511	S77			
	D512	S30			
	T513	S27			
**p72 in complex with F11**	Q123	V104			
	G124			Q52	
	R139	E112		Y51	
	N140	D32			
	L157	Y56			
	I165	Y56			
	V166	L105			
	P167	Y56	L105		
	R511	S77	K79		
	T513	S30			
	A514	S30			
**p72 in complex with C9**	Q123			N53	
	R139			R55	
	N140	S103		N51	
	Y142	W100		Y50	
	P149	W100			
	R385			R94	
	Q509			R94	
	D510	R57			
	R511			N97	
	D512			R94	
	A514			Y33	
	L515			Y33	

**p72 in complex with H3**	R139		R56	T58
	N140		N52	
	P149	W100		
	Y142	W100		
	L150	W100		
	G152	C101		
	D510	R57		
	R511	Y59		
	D512	T104		
	T513	T103		
